# A Comprehensive Review of Proximal Humerus Fractures: From Epidemiology to Treatment Strategies

**DOI:** 10.7759/cureus.57691

**Published:** 2024-04-05

**Authors:** Saksham Goyal, Ratnakar Ambade, Rahul Singh, Ashutosh Lohiya, Hardik Patel, Siddharth K Patel, Kashyap Kanani

**Affiliations:** 1 Orthopaedics, Jawaharlal Nehru Medical College, Datta Meghe Institute of Higher Education & Research, Wardha, IND

**Keywords:** imaging, treatment, classification, scores, proximal humerus fractures

## Abstract

This comprehensive review delves into the intricate landscape of proximal humerus fractures (PHFs), exploring their epidemiology, historical evolution, contemporary classification systems, treatment strategies, and outcome measures. PHFs present a complex orthopedic challenge, necessitating a nuanced understanding of their multifaceted dimensions. Despite their clinical significance, PHFs remain relatively understudied in population-based epidemiology. This review critically examines existing literature to uncover the incidence, prevalence, and demographic patterns associated with these fractures. A foundational understanding of the epidemiological landscape is crucial for effective preventive strategies and optimized fracture management. Tracing back to historical records, the review explores the evolution of diagnostic and therapeutic approaches for PHFs. From ancient treatment modalities documented on the Edwin Smith papyrus to contemporary X-ray-based classifications such as Neer and AO/OTA, a historical context is provided to understand the journey of managing these fractures. Navigating through a spectrum of treatment strategies, the review contrasts nonoperative approaches with various surgical interventions. The challenges and outcomes associated with conservative management are juxtaposed against methods like open reduction internal fixation and tension band osteosynthesis. Evidence synthesis guides clinicians in making informed decisions based on patient characteristics and fracture complexities. Central to assessing PHF management are patient-reported outcome measures. The review explores the significance of instruments such as the Disabilities of the Arm, Shoulder, and Hand (DASH) questionnaire and the Constant-Murley score in evaluating treatment success. The shift toward subjective measures is discussed, considering their correlation with patient experiences and the concept of minimal clinically important difference. The impact of demographic factors, including age and gender, on PHFs is scrutinized. The association between these fractures and osteoporosis is highlighted, emphasizing the crucial role of bone health in fracture prevention and management. Through this comprehensive exploration, the review provides a robust foundation for understanding, evaluating, and advancing the management strategies for PHFs. The synthesis of historical perspectives, contemporary classifications, and treatment modalities serves as a valuable resource for the orthopedic community, fostering improved clinical decision-making and patient outcomes.

## Introduction and background

Proximal humerus fractures (PHFs), characterized by their intricate nature and clinical significance, stand as a formidable challenge in orthopedic practice. This introduction lays the groundwork for an in-depth exploration of the multifaceted landscape surrounding PHFs, aiming to provide a comprehensive perspective for healthcare professionals. PHFs represent the third most common osteoporotic fracture among the aging population, following hip and distal radius fractures, which constitute 4% of all fractures [1-3]. Over 70% of patients with PHFs are aged 60 or older, with women comprising 75% of this demographic [4]. In elderly individuals, low-energy injuries, typically falling from a standing height, account for the majority of cases [5]. Over the previous 33 years, hospitalizations for PHFs have increased by 13% per year, according to a thorough analysis based on hospital discharge data. In 2002, the incidence of these cases rose to 105 per 100,000 person-years [6]. Currently, only incomplete and inconclusive data are available on the actual incidence of PHF, describing preselected cohorts with observed incidences ranging from 60.1 to 90.8 per 100,000 person-years. There is insufficient information to establish the optimal course of treatment, even though 85% of PHFs are nondislocated and handled conservatively [7]. PHFs, intricate in their presentation and management, demand meticulous attention. Their unique challenges necessitate a nuanced understanding, spanning from diagnosis and classification to evolving treatment strategies. Despite their prevalence, a detailed exploration is warranted to enhance the precision of fracture management. This review seeks to dissect various dimensions of PHFs, offering insights into their epidemiology, historical evolution, contemporary treatment modalities, and outcome measures. The overarching goal is to equip healthcare professionals with a nuanced understanding to enhance decision-making and optimize patient outcomes. This review delves into the diverse treatment strategies available for PHFs. Whether considering nonoperative approaches or exploring surgical interventions like open reduction and internal fixation (ORIF), the synthesis of evidence aims to guide clinicians in making informed decisions tailored to individual patient needs. Central to evaluating PHF management are patient-reported outcome measures (PROMs). The review scrutinizes the significance of instruments like the Disabilities of the Arm, Shoulder, and Hand (DASH) questionnaire, emphasizing the shift toward subjective measures and their correlation with patient experiences. Understanding the influence of demographic factors and their association with osteoporosis on PHFs is pivotal. Age, gender, and bone health intricately shape the fracture landscape, allowing for a more nuanced and personalized approach to fracture prevention and management. While some studies have explored various treatment methods, the overall quality has been suboptimal, with a scarcity of high-quality randomized controlled trials (RCTs) comparing surgical and nonsurgical approaches [8-10]. Differentiating outcomes between working-age and elderly patients is essential, yet previous studies have predominantly relied on range of motion (ROM) as an outcome measure, which inadequately captures functional aspects like strength, pain, and patient satisfaction [8].

In synthesizing these facets, this comprehensive review endeavors to offer a robust resource for the orthopedic community. By weaving together historical insights, contemporary perspectives, and a nuanced understanding of PHFs, this review aims to enhance clinical decision-making and elevate patient outcomes.

## Review

Incidence

Despite the prevalence of PHFs in clinical practice, the epidemiological landscape surrounding this condition remains surprisingly underexplored. Only a handful of population-based studies have delved into a comprehensive understanding of PHFs, encompassing both inpatient and outpatient treatments. Between 2016 and 2018, the unadjusted incidence rate in the Spanish city of Vigo was 60.1 per 100,000 inhabitants per year [11]. This indicates a significant burden of PHFs within the community, emphasizing the need for further research and attention to this orthopedic concern. It is crucial to approach PHF incidence figures with caution, recognizing that unadjusted incidences are not universally applicable to different populations or periods within the same geographical area. Moreover, the methodology employed in data collection plays a pivotal role in shaping incidence figures. Studies relying on hospital discharge registers may underestimate the total incidence rate [12].

Etiology and risk factors

The etiology of PHFs is intricate, often arising from a combination of intrinsic and extrinsic factors. Age is a prominent factor, with approximately 90% of these fractures occurring in the elderly, particularly those over the age of 60 [3]. The natural decrease in bone density associated with aging predisposes older individuals to fractures, especially in scenarios involving low-energy trauma [4]. Osteoporosis, characterized by reduced bone mineral density, significantly amplifies the risk of PHFs, given the weakened state of bones, a prevalent condition in the aging population [13].

Falls are a primary cause of PHFs, especially in the elderly. Whether from a standing height or involving an outstretched arm, falls can result in fractures due to compromised bone strength, further emphasizing the relationship between age and fracture susceptibility [14]. While high-energy trauma, such as motor vehicle accidents or sports injuries, can lead to PHFs in younger individuals, older individuals are susceptible even to low-energy trauma due to diminished bone strength.

Gender differences play a role, with women, particularly postmenopausal women, exhibiting a higher prevalence of PHFs. The decline in estrogen levels after menopause contributes to decreased bone density, elevating the risk of fractures. Underlying bone health disorders, including osteopenia and osteomalacia, heighten the susceptibility to fractures by compromising bone integrity [15]. Conditions like rheumatoid arthritis or other inflammatory joint disorders can impact the structural integrity of the proximal humerus, as can preexisting rotator cuff pathology, such as degenerative changes or tears, which may compromise shoulder joint stability.

Chronic medical conditions and certain medications, such as long-term corticosteroid use, are additional factors influencing fracture risk [16]. Smoking, identified as a risk factor for PHFs, may contribute to compromised bone health and delayed fracture healing. Understanding these multifaceted risk factors is essential for developing preventive strategies and providing comprehensive care for individuals vulnerable to PHFs.

Clinical presentation

The clinical presentation of PHFs can vary based on the severity of the injury, the specific fracture pattern, and individual patient factors. Typically, patients with PHFs exhibit a combination of symptoms and physical findings that aid in the diagnosis. A hallmark symptom of PHF is pain in the shoulder region. The intensity of pain can vary, often worsening with movement or palpation of the affected area. Swelling around the shoulder joint is a common occurrence after a PHF, accompanied by bruising (ecchymosis), particularly in the days following the injury.

Patients may experience a significant reduction in the ROM of the affected shoulder. Both active and passive movements can be restricted due to pain and mechanical blockage caused by the fracture. In more severe fractures, a visible deformity or displacement of the shoulder may be apparent, allowing for observation by comparing the injured shoulder to the unaffected side. Palpation of the proximal humerus elicits tenderness at the fracture site, aiding in identifying the specific location and severity of the fracture. Crepitus, a grating or grinding sensation, may be felt or heard during shoulder movements, indicating bone fragments rubbing against each other.

In some cases, neurovascular symptoms may be present, manifesting as numbness, tingling, or weakness in the arm or hand, suggesting potential nerve involvement. The axillary nerve is most commonly affected in PHFs [17]. Patients often instinctively guard the affected shoulder, limiting movement to minimize pain. Splinting, or holding the arm close to the body, is a common protective mechanism observed in individuals with PHFs. This comprehensive clinical presentation provides valuable insights for healthcare professionals in diagnosing and managing PHFs.

Diagnostic imaging and techniques

Imaging studies play a pivotal role in confirming the diagnosis of PHFs and providing detailed information about the fracture pattern. Standard X-rays, including anteroposterior, lateral, and axillary views, are typically the initial imaging modalities used to assess the shoulder. X-rays offer crucial insights into the alignment and integrity of the proximal humerus and provide essential information about the number of parts involved, displacement, and angulation of the fracture fragments [18].

In cases involving complex fractures or when detailed visualization is necessary, CT scans may be utilized. CT scans provide three-dimensional views of the fracture, offering a more comprehensive understanding of the fracture configuration. This is particularly valuable for surgical planning in intricate cases [18]. While MRI is less commonly used for routine fractures, it may be employed when soft tissue injuries or associated injuries, such as rotator cuff tears, are suspected. MRI provides detailed images of soft tissues and aids in assessing the extent of soft tissue damage [19]. Ultrasonography may be used in specific situations to evaluate soft tissue injuries, such as tendon or ligament damage [20]. However, it is not the primary imaging modality for diagnosing PHFs.

These imaging studies are essential not only for confirming the diagnosis but also for determining the most appropriate course of treatment. They guide orthopedic surgeons in choosing between conservative management and surgical intervention based on the fracture’s characteristics [21]. Additionally, imaging aids in assessing potential complications, such as avascular necrosis, that may impact the overall prognosis and management strategy.

Classifications

PHFs exhibit diverse presentations, necessitating a sophisticated classification system for effective treatment guidance. Over the years, several classification systems have emerged, each offering a unique perspective on fracture patterns. The historical context includes the ancient Edwin Smith papyrus, dating back to 1600 BCE, which outlined basic treatments like bandaging and traction reduction [22]. In the 19th century, early classifications focused on bandaging and traction, yielding unsatisfactory outcomes.

Modern classification systems include the Neer classification, introduced in 1970 by Charles Neer, which categorizes fractures based on the number of parts involved and the displacement of fragments [23]. It remains widely used, defining one- to four-part fractures based on the involvement of the humeral head, surgical neck, greater tuberosity, and anatomical neck, respectively. The AO/OTA classification, developed in 1987 by Müller et al., offers a more detailed categorization based on anatomical location, configuration, and severity, but its complexity limits routine clinical use [24]. Modern X-ray-based classification systems, such as AO/OTA and Neer, dominate contemporary practice. Neer’s 6-phase classification further divides the proximal humerus into four components, each indicating the level of fracture involvement [14]. Additional classifications, like Codman-Hertel and Resch, introduce simplicity or include various fracture types, respectively [25].

The results of the Neer classification system show a moderate degree of agreement, with four-part fractures and an interobserver mean kappa value of 0.48. Fair to moderate agreement was shown in the Codman-Hertel classification, which achieved a kappa value of 0.44 in an interobserver analysis using plain X-rays [26]. Regardless of the classification system employed, these data demonstrate the low degree of agreement among surgeons when it comes to fracture categorization. On the other hand, systematic classification training improves classification agreement and prediction value [27]. While CT and three-dimensional CT are useful for preoperative planning, they did not appear to have a greater impact on the categorization of displaced fractures.

Treatment

Nonoperative Treatment

Nonoperative management of PHFs has been a longstanding approach, historically employed for minimally displaced fractures or in elderly patients with lower functional demands. While still relevant for specific cases, it faces challenges in maintaining reduction for complex fractures, and there is a potential for malunion or nonunion. The nonoperative approach is commonly considered for fractures with minimal displacement, particularly in older adults. However, its application is limited to more complex fractures, and the decision to opt for nonoperative management is individualized based on fracture characteristics and patient factors [14]. In a retrospective comparison study conducted in 2023, involving 160 patients with PHFs (mean age: 71 years), 54 patients underwent nonoperative care, while 106 patients opted for surgery. The follow-up period extended to two years, and outcomes were assessed using the DASH score. At the end of the trial, no significant difference was observed between operative treatment and nonoperative treatment [10]. Another retrospective analysis, focusing on impacted varus fractures in 2004, opted for nonoperative treatment. According to the Neer score, 79% of the 99 patients in this trial who had a one-year follow-up reported good or excellent results [3]. The same research group’s 2002 publication noted that outcomes were adversely affected by growing older and more displaced. Based on X-ray analysis, 160 patients (mean age: 63 years) who were not surgically treated had more than half of them with two-part (38%) or three-part (14%) fractures. After a year, the group that was not sick had a 10.2-point difference in the DASH score and an 8.2-point difference in the Constant-Murley score. While the constant values were below the minimal clinically important difference, the DASH score marginally exceeded it. Smokers had a 5.5-fold higher chance of nonunion status than nonsmokers, with the likelihood of nonunion status being 7% [28-30].

Surgical Treatment

Closed reduction and percutaneous pinning (CRPP): CRPP is a surgical technique used for certain types of fractures, particularly two-part fractures where the bone is broken into two main pieces. This method involves manipulating the fractured bones back into their proper alignment without making a large incision (closed reduction) and then stabilizing them with K-wires inserted through the skin (percutaneous pinning). It is often chosen for specific fracture patterns like two-part PHFs (those occurring near the shoulder), ideally at the surgical neck (the area just below the ball of the shoulder joint), as well as for three- or four-part fractures where there is enough bone remaining for the pins to hold onto. However, there are limitations to its effectiveness, especially in cases where there is significant displacement (bones are not in proper alignment) or comminution (the bone is broken into many pieces). In a study involving 51 patients with significant underlying health issues, with an average age of 76 years, who were not suitable candidates for ORIF, 23 were treated with braces (a nonsurgical approach), and 28 underwent CRPP. The results were compared between the two groups: the surgical group (CRPP) had a constant score of 81 out of 100, which is a measure of shoulder function and strength; a higher score indicated a better outcome; and a VAS score of 2.9 out of 10, which measures pain on a scale from 0 to 10, with lower scores indicating less pain. This suggests that CRPP provided good functional outcomes and pain relief compared to the nonsurgical group.

Helix wire: The introduction of helix wire as a minimally invasive instrument marked a notable development in the management of fractures. Through a “screwing” mechanism, titanium helical wire injected via the diaphysis served to stabilize fractures. Early studies were encouraging, showing good to exceptional results, particularly in instances with two- and three-part fractures [31-33]. Nonetheless, the general agreement indicates that this method produces less than ideal outcomes, with correlations to primary failure and nonunion rates as high as 47% [34,35]. Surprisingly, nothing fresh has been published on helix wire since 2010.

Tension band technique: This method impacts the humeral head and shaft manually using a deltopectoral approach. Lag screw fixation is accomplished using a 6.5-mm AO screw, and two 18-gauge stainless steel wires are inserted through drill holes beneath the rotator cuff and fastened to the shaft distally [36]. A total of 35 patients with four-part fractures received ORIF using a tension band and K-wires. Out of the 21 instances (64%), the results indicated excellent and good outcomes, while 12 cases had nonsatisfactory or bad outcomes. In 27% of the patients, avascular necrosis was noted [37]. A follow-up RCT was done in 1997 to examine the use of tension bands in the management of PHFs. Two groups of 40 patients with three- or four-part fractures were randomly assigned to receive tension band osteosynthesis treatment, and another group was managed conservatively. Major complications were noted in the patients treated surgically. A radiological evaluation revealed that, although the fractured humeral head’s location had improved due to surgery, function had not improved [38].

External fixation: External fixation may be used for temporary stabilization in polytrauma or severely comminuted fractures. Its use is limited due to associated complications, including pin site infections and reduced patient comfort. The first randomized trial on proximal humeral fractures was conducted in Denmark, marking a turning point in the field’s understanding of fracture care. The patients in this consecutive series had displaced two- to four-part fractures, and their ages ranged from 30 to 91 years. Randomization was used to assign them to have closure reduction (n = 15, median age: 72 years) or Hoffman’s external fixator (n = 16, median age: 66 years). When compared to closed reduction, the one-year follow-up showed that external fixation produced better results. However, direct comparisons with more current studies are limited by the use of an antiquated outcome score (Neer score). The study’s statistical strength was weakened by the tiny group sizes, even with the current approach [39]. The same authors also described the surgical method in another paper that included early findings from a small pilot group [40].

Plating: Plating techniques for PHFs evolved from early innovations in internal fixation during the 20th century. The primary goal of plating is to achieve stable fixation, facilitate early mobilization, and minimize complications. Locking plates, introduced to address issues of screw purchase in osteoporotic bone, have become popular. While locking plates provide improved stability, nonlocking plates may be preferred in certain scenarios, as a nonlocking plate obtains fixation stability through the frictional force between the plate and bone. One-third of tubular plates, J-plates, and T-plates were among the nonlocking plates that were in use before the introduction of locking plates. However, frequent side effects that altered the course of treatment included avascular necrosis, infections, fixation loosening, and reoperations [41]. The first locking plates were made available in 2001 [7]. One such plate is the Proximal Humerus Internal Locking System (PHILOS) plate. It is characterized by angular stability, providing enhanced support in the inherently unstable proximal humerus region. It utilizes locking screw technology, allowing screws to engage with the plate at fixed angles for improved construction stability. Designed to closely match the anatomy of the proximal humerus, it facilitates optimal placement and fixation [42]. Surgical fixation with the PHILOS plate can be accomplished through minimally invasive or open approaches, providing flexibility to surgeons. The PHILOS plate is suitable for a variety of PHF types, including two-part, three-part, and certain four-part fractures. It is particularly advantageous in osteoporotic bone, where traditional fixation methods may be less reliable. Additionally, it is effective in managing head-splitting fractures, contributing to anatomical reduction and stability. The implant is made to reduce interference with the humeral head’s blood supply to promote improved results. However, despite its advantages, screw cutouts can occur [43].

Intramedullary nails: Because intramedullary devices, such as nails, only require a minor incision for fracture repair, it is thought that their use reduces surgical trauma. With their proximal insertion through the rotator cuff, these nails are secured with screws in both the proximal and distal directions, providing enhanced stiffness in the spaces between fracture pieces in more sophisticated designs. Notwithstanding their possible benefits, there is a paucity of comparative trials and outcome research. Problems were linked to a notably high prevalence of 28% in a short retrospective cohort study with 27 participants, despite a mean constant score of 75 [44]. The new straight nail, MultiLoc Proximal Humeral Nail (MPHN; MultiLoc PHAN, Synthes GmbH, Solothurn, Switzerland], offers a variety of locking options. By creating a safe zone between the lateral head fracture line and the nail insertion hole in the head segment, the straight design is said to improve the stability of the proximal nail end and stop an uncontrolled crack in this location. For unstable PHFs, the “screw in screw” approach may be useful in reducing the likelihood of secondary loss of reduction. It accomplishes this by giving the medial hinge more support and permitting more proximal fixation in the humeral head’s posteromedial region [45]. Two antegrade intramedullary nail treatments were randomly assigned to 54 patients with displaced Neer two- or three-part PHFs: 28 patients had therapy with the MPHN, and 26 patients received treatment with the Polarus humeral nail. The study found that patients treated with MPHN had improved functional outcomes, primarily in terms of pain, and that there were no statistically significant differences in the final functional outcomes between the two nails (mean adjusted constant score: 73 in the Polarus nail and 83 in the MPHN nail) [46].

Hemiarthroplasty (HA): HA, replacing the humeral head with a prosthetic implant, has been employed for severe fractures with compromised vascularity. In situations where ORIF is not feasible, such as severe osteoporosis, a head impression, or a split fracture, HA is a commonly employed approach for comminuted fractures [47]. Although it is effective for pain relief, it sacrifices joint preservation and may not be suitable for more active individuals. Making sure the tuberculum recovers in an anatomical position to support appropriate rotator cuff function is a crucial part of HA. Up to 30% of tuberosity resorption can hurt outcome measures [48]. When displaced four-part fractures in older adults were compared to nonoperative and HA treatments, the first RCT revealed that arthroplasty provided a clinically meaningful improvement in quality of life. Pain relief was the main advantage of HA, but functional results were unaffected. A total of 23 patients were recruited: 12 were managed with HA, and 11 had reverse total shoulder arthroplasty (rTSA). The limitations were that a longer follow-up would have been preferred. The mean follow-up was 3.6 years, making it the longest comparative study. Other limitations were the retrospective nature of the trial and surgeon preference toward rTSA in older patients [49]. Another trial with patients with four-part fractures compared HA with nonoperative treatment; no differences were observed in functional outcomes. A total of 50 patients were recruited and were managed with either of the treatments. The limitation of this study was the low number of patients and only 12 months of follow-up [9].

rTSA: rTSA, reversing the ball-and-socket configuration, is often chosen for elderly patients with rotator cuff deficiency. While addressing rotator cuff insufficiency, rTSA involves a more complex surgical approach and has considerations for implant longevity. As the name suggests, the prosthesis aims to reverse the normal anatomy, where the scapula articulates with a glenoid cavity and the humerus has a spherical head. Following a reverse prosthesis, the humerus has a concave counter-articulation, and the scapula is linked to the spherical articulation. By laterally repositioning the rotational center and introducing offset, the primary objective is to enhance deltoid muscle activation and movement momentum, eliminating the dependency on the rotator cuff. Nine patients in each group had three or four component fractures, and the results after a year of follow-up showed no significant differences among the 21 patients treated with ORIF, HA, or reverse shoulder arthroplasty (RSA). The RSA group exhibited a greater number of patients who were able to attain forward flexion of more than 90°, suggesting that this approach may have advantages. In a different comparative study, after receiving treatment with either HA or RSA for at least two years, 53 consecutive patients (mean age: 74 years) demonstrated better functional outcomes, as indicated by the scores on the Simple Shoulder Test (HA 5.8 vs. RSA 7.7) and the American Shoulder and Elbow Surgeons (ASES) (HA 62 vs. RSA 77). This information was based on the RSA group. These findings suggest that RSA may have benefits in a few functional areas, which should be considered when choosing a surgical treatment for fractures of the proximal humerus. In one ongoing RCT, RSA is being used to compare a locking plate. The results have not been confirmed in their entirety [50].

Emerging techniques

Biological augmentation involves the exploration of using biological agents, such as growth factors or stem cells, to enhance the healing process by stimulating bone formation and accelerating the regeneration of damaged tissues [51]. Three-dimensional printing technology has revolutionized medical interventions by enabling the creation of patient-specific implants tailored to the unique anatomy of individuals. This personalized approach enhances implant fit, potentially reducing complications and improving overall outcomes [52]. In the realm of augmented reality and virtual reality, these technologies are increasingly integrated into preoperative planning. Surgeons are provided with immersive experiences for visualizing fracture patterns and practicing procedures, ultimately aiding in enhancing surgical precision [53]. Nanotechnology is leveraged in creating coatings for implants, specifically in the domain of implant coatings. This application of nanotechnology promotes improved biocompatibility and reduces the risk of complications. Nanostructured surfaces created through nanotechnology may enhance osseointegration [54].

Rehabilitation and post-treatment care

Rehabilitation and post-treatment care are integral components of optimizing outcomes for patients recovering from PHFs. This multifaceted approach encompasses various aspects, including pain management, the restoration of ROM, rebuilding strength, and ensuring a gradual return to functional activities. During the early postoperative period, effective pain control measures are initiated, considering the patient’s comfort and overall well-being. This may involve the use of medications, physical modalities, and ice applications. Techniques to manage postoperative swelling, such as elevation and compression, are employed to minimize discomfort and enhance the healing process.

Passive ROM exercises are initiated early on to prevent joint stiffness. These exercises are carefully designed to avoid stressing the healing structures while promoting joint flexibility. Immediately after the operation, the patient is advised to do shoulder shrugging exercises and elbow ROM exercises to prevent elbow stiffness. After three to four weeks, the patient is started on shoulder ROM exercises. As healing progresses, patients transition to active-assisted ROM exercises, guided by therapists through controlled movements, to gradually improve shoulder mobility [55]. Special attention is given to scapular mobility exercises, addressing the interplay between the shoulder and scapula for optimal function. The rehabilitation process also includes a progression from isometric exercises, initially introduced to activate shoulder muscles without excessive joint stress, to resistance training as tolerated. This targets specific muscle groups involved in shoulder stability and function. Functional training becomes a key focus as rehabilitation advances, incorporating task-specific activities that mirror daily tasks. This may involve reaching, lifting, and overhead activities to simulate real-world demands. The rehabilitation plan is tailored to each patient’s functional goals and lifestyle demands, guiding them through the progressive reintegration of activities of daily living (ADLs).

Outcome measures

In the early 1900s, a study recommended post-therapy maintenance and treatment evaluation, a concept that was later widely accepted. However, it took more than 80 years for the development of the Constant-Murley score, marking the emergence of the first numerical result assessment system for the shoulder [56]. Chronic pain after trauma or surgery is acknowledged to have a deleterious effect on an individual’s quality of life [57]. The Visual Analog Scale (VAS) stands out as the most efficient, dependable, and user-friendly method for measuring pain in clinical settings [58]. Pain and ROM were initially considered the most crucial outcome indicators in PHFs before the widespread use of the constant score [59]. Health-related quality of life (HRQOL) measures play a pivotal role in assessing the overall well-being of individuals, encompassing physical, social, emotional, and cognitive dimensions. EuroQol (EQ-5D) and 15D are widely used in PHFs, with good responsiveness reported, although some studies note a significant ceiling effect, where scores cluster at the upper limit, diminishing sensitivity [60]. Scoring systems are indispensable tools for evaluating the functional outcomes and overall success of interventions in PHFs. Various scoring systems provide a standardized framework to quantify aspects such as pain, function, and patient satisfaction.

The Neer score, developed by Dr. Charles Neer, categorizes outcomes based on pain relief, function, and patient satisfaction, considering criteria like pain relief, ROM, strength, and radiographic parameters [61]. The Constant-Murley score, introduced by Cristopher Constant and Alan Murley, evaluates both subjective and objective parameters, including pain, ADLs, ROM, and strength assessments [62]. Despite their labor-intensive nature, subjective surveys are often chosen over the constant score due to the demonstrated correlation between them [62]. The DASH score, a PROM, emphasizes the impact on daily activities, encompassing physical function, symptoms, emotional impact, and social functioning [63]. The DASH questionnaire assesses patient happiness and function on a scale from 1 (normal limb usage) to 5 (nonfunctional), depending on specific tasks [63]. A quick DASH, made up of 11 questions from the original DASH, was created in recognition of its potential complexity [64]. The Oxford Shoulder Score, developed by Dawson et al. in 1996, is a subjective, shoulder-specific score that assesses shoulder problems based on 12 items about daily activities [65]. The ASES score, specifically designed for shoulder pathologies, including PHFs, combines physician-assessed and patient-reported components for a comprehensive evaluation [66]. The Rowe score, particularly prevalent in research studies, assesses shoulder function post-treatment, evaluating criteria such as pain, ROM, strength, and joint stability [67]. The VAS for pain, a subjective pain assessment tool, utilizes a linear scale ranging from 0 (no pain) to 100 (worst pain imaginable) and is widely employed for pain measurement during rest, movement, and specific activities [58].

These scoring systems collectively serve as valuable instruments for evaluating treatment efficacy and patient-reported outcomes following PHFs. Clinicians often employ a combination of these tools to comprehensively assess the multifaceted nature of recovery, allowing for tailored interventions and improved patient care.

Challenges

Addressing PHFs involves navigating challenges that span diagnostic intricacies to evolving treatment paradigms. The diagnostic complexity associated with these fractures is particularly pronounced, making an accurate diagnosis, especially in distinguishing between fracture types, a challenging endeavor. The management of PHFs is further complicated by the diverse spectrum of fracture patterns, necessitating individualized treatment decisions. The ongoing debates between nonoperative and operative approaches underscore the absence of a one-size-fits-all solution [68]. The link between osteoporosis and PHFs adds another layer of complexity to their management. Effectively addressing bone health is integral but proves to be challenging, particularly in the elderly population, where osteoporosis is often a significant concern [13].

Complications pose a substantial threat to treatment success, with avascular necrosis being a notable example. Additionally, nonunion rates, especially in certain fracture types, remain a significant concern, emphasizing the need for careful consideration and management of potential complications [69]. Furthermore, the reliance on subjective measures, such as patient-reported outcomes, introduces variability in the assessment process. It is recognized that these patient-reported outcomes may not always align entirely with clinical assessments, highlighting the importance of adopting a holistic approach to evaluating treatment success and patient well-being. Understanding and addressing these challenges are crucial for enhancing patient outcomes in the management of PHFs [68].

Future directions

Continued research is underway to advance imaging modalities, aiming for improved accuracy in preoperative planning. One avenue being explored involves the integration of artificial intelligence and machine learning into diagnostic processes, with the potential to enhance diagnostic precision significantly [70]. In the realm of treatment approaches for PHFs, there is a discernible shift toward developing more personalized treatment algorithms. These algorithms are tailored to individual patients, taking into account specific factors that can influence the most effective course of action.

Biological interventions are also gaining attention as potential strategies to promote fracture healing. Growth factors, stem cell therapy, and tissue engineering are among the biological approaches being explored in the context of PHFs, with the aim of enhancing the body’s natural healing processes [51]. In parallel, there is a growing emphasis on the development of comprehensive rehabilitation protocols. These protocols go beyond addressing merely the physical aspects of recovery and extend to encompass psychosocial considerations. The goal is to create rehabilitation strategies that holistically support patients in their journey toward optimal recovery [51].

## Conclusions

PHFs in the elderly pose common challenges, and their management depends on the extent of displacement. While minimally displaced fractures can often be treated conservatively with early physical therapy, displaced fractures require careful consideration of various factors. When determining the appropriate treatment for displaced fractures, the patient’s level of independence, bone quality, and surgical risk factors should be taken into account. Several acceptable treatment options include percutaneous techniques, intramedullary nails, locking plates, and arthroplasty. Internal fixation methods demand special attention to specific factors. Medial comminution, varus angulation, and the restoration of the calcar are critical considerations for successful outcomes. The goal is to achieve stability and proper alignment for optimal healing. Alternatives such as HA and RSA have proven to be effective, but concerns persist, particularly regarding tuberosity nonunion with HA. Ultimately, the choice of treatment modality should be individualized based on a thorough assessment of the patient’s overall health, lifestyle, and specific fracture characteristics. Regular follow-up and rehabilitation play crucial roles in achieving the best possible outcomes for elderly patients with PHFs. To establish the optimal treatment method for PHFs, RCTs and cost analyses are imperative. These studies can provide valuable insights into the comparative effectiveness, safety, and economic implications of various treatment approaches. By weighing the benefits and drawbacks of each method through rigorous research, healthcare professionals can make more informed decisions tailored to individual patient needs, ultimately improving overall outcomes in the management of PHFs.

## References

[1] Browner WS, Seeley DG, Vogt TM, Cummings SR (1991). Non-trauma mortality in elderly women with low bone mineral density. Lancet.

[2] Lauritzen JB, Schwarz P, Lund B, McNair P, Transbøl I (1993). Changing incidence and residual lifetime risk of common osteoporosis-related fractures. Osteoporos Int.

[3] Court-Brown CM, Caesar B (2006). Epidemiology of adult fractures: a review. Injury.

[4] Court-Brown CM, Garg A, McQueen MM (2001). The epidemiology of proximal humeral fractures. Acta Orthop Scand.

[5] Panula J, Pihlajamäki H, Mattila VM, Jaatinen P, Vahlberg T, Aarnio P, Kivelä SL (2011). Mortality and cause of death in hip fracture patients aged 65 or older - a population-based study. BMC Musculoskelet Disord.

[6] Palvanen M, Kannus P, Niemi S, Parkkari J (2006). Update in the epidemiology of proximal humeral fractures. Clin Orthop Relat Res.

[7] Bell JE, Leung BC, Spratt KF, Koval KJ, Weinstein JD, Goodman DC, Tosteson AN (2011). Trends and variation in incidence, surgical treatment, and repeat surgery of proximal humeral fractures in the elderly. J Bone Joint Surg Am.

[8] Lanting B, MacDermid J, Drosdowech D, Faber KJ (2008). Proximal humeral fractures: a systematic review of treatment modalities. J Shoulder Elbow Surg.

[9] Boons HW, Goosen JH, van Grinsven S, van Susante JL, van Loon CJ (2012). Hemiarthroplasty for humeral four-part fractures for patients 65 years and older: a randomized controlled trial. Clin Orthop Relat Res.

[10] Launonen AP, Sumrein BO, Reito A (2023). Surgery with locking plate or hemiarthroplasty versus nonoperative treatment of 3-4-part proximal humerus fractures in older patients (NITEP): an open-label randomized trial. PLoS Med.

[11] Iglesias-Rodríguez S, Domínguez-Prado DM, García-Reza A, Fernández-Fernández D, Pérez-Alfonso E, García-Piñeiro J, Castro-Menéndez M (2021). Epidemiology of proximal humerus fractures. J Orthop Surg Res.

[12] Palvanen M, Kannus P, Niemi S, Parkkari J (2010). Secular trends in distal humeral fractures of elderly women: nationwide statistics in Finland between 1970 and 2007. Bone.

[13] Oh JH, Song BW, Lee YS (2014). Measurement of volumetric bone mineral density in proximal humerus using quantitative computed tomography in patients with unilateral rotator cuff tear. J Shoulder Elbow Surg.

[14] Schumaier A, Grawe B (2018). Proximal humerus fractures: evaluation and management in the elderly patient. Geriatr Orthop Surg Rehabil.

[15] Föger-Samwald U, Dovjak P, Azizi-Semrad U, Kerschan-Schindl K, Pietschmann P (2020). Osteoporosis: pathophysiology and therapeutic options. EXCLI J.

[16] Panday K, Gona A, Humphrey MB (2014). Medication-induced osteoporosis: screening and treatment strategies. Ther Adv Musculoskelet Dis.

[17] Visser CP, Coene LN, Brand R, Tavy DL (2001). Nerve lesions in proximal humeral fractures. J Shoulder Elbow Surg.

[18] Rudran B, Little C, Duff A, Poon H, Tang Q (2022). Proximal humerus fractures: anatomy, diagnosis and management. Br J Hosp Med (Lond).

[19] Voigt C, Ewig M, Vosshenrich R, Lill H (2010). Value of MRI in preoperative diagnostics of proximal humeral fractures compared to CT and conventional radiography [Article in German]. Unfallchirurg.

[20] Ackermann O, Sesia S, Berberich T (2010). Sonographic diagnostics of proximal humerus fractures in juveniles [Article in German]. Unfallchirurg.

[21] Cancio-Bello AM, Barlow JD (2023). Avascular necrosis and posttraumatic arthritis after proximal humerus fracture internal fixation: evaluation and management. Curr Rev Musculoskelet Med.

[22] Brorson S (2009). Management of fractures of the humerus in Ancient Egypt, Greece, and Rome: an historical review. Clin Orthop Relat Res.

[23] Carofino BC, Leopold SS (2013). Classifications in brief: the Neer classification for proximal humerus fractures. Clin Orthop Relat Res.

[24] Helfet DL, Haas NP, Schatzker J, Matter P, Moser R, Hanson B (2003). AO philosophy and principles of fracture management-its evolution and evaluation. J Bone Joint Surg Am.

[25] Majed A, Macleod I, Bull AM (2011). Proximal humeral fracture classification systems revisited. J Shoulder Elbow Surg.

[26] Brorson S, Bagger J, Sylvest A, Hrobjartsson A (2009). Diagnosing displaced four-part fractures of the proximal humerus: a review of observer studies. Int Orthop.

[27] Brorson S, Bagger J, Sylvest A, Hróbjartsson A (2002). Low agreement among 24 doctors using the Neer-classification; only moderate agreement on displacement, even between specialists. Int Orthop.

[28] Court-Brown CM, Cattermole H, McQueen MM (2002). Impacted valgus fractures (B1.1) of the proximal humerus. The results of non-operative treatment. J Bone Joint Surg Br.

[29] Court-Brown CM, McQueen MM (2004). The impacted varus (A2.2) proximal humeral fracture: prediction of outcome and results of nonoperative treatment in 99 patients. Acta Orthop Scand.

[30] Fjalestad T, Strømsøe K, Blücher J, Tennøe B (2005). Fractures in the proximal humerus: functional outcome and evaluation of 70 patients treated in hospital. Arch Orthop Trauma Surg.

[31] Carbone S, Tangari M, Gumina S, Postacchini R, Campi A, Postacchini F (2012). Percutaneous pinning of three- or four-part fractures of the proximal humerus in elderly patients in poor general condition: MIROS® versus traditional pinning. Int Orthop.

[32] Gorschewsky O, Puetz A, Klakow A, Pitzl M, Neumann W (2005). The treatment of proximal humeral fractures with intramedullary titanium helix wire by 97 patients. Arch Orthop Trauma Surg.

[33] Krause FG, Gathmann S, Gorschewsky O (2008). The use of intramedullary helix wire for the treatment of proximal humerus fractures. J Orthop Trauma.

[34] Raissadat K, Struben PJ, van Loon CJ (2004). Helix wire osteosynthesis for proximal humeral fractures: unacceptable nonunion rate in two- and three-part fractures. Arch Orthop Trauma Surg.

[35] Khan SA (2008). Helix wire osteosynthesis of proximal humerus fractures: unacceptably high rate of failure. Acta Orthop Belg.

[36] Cornell CN (1994). Tension-band wiring supplemented by lag-screw fixation of proximal humerus fractures: a modified technique. Orthop Rev.

[37] Darder A, Darder A Jr, Sanchis V, Gastaldi E, Gomar F (1993). Four-part displaced proximal humeral fractures: operative treatment using Kirschner wires and a tension band. J Orthop Trauma.

[38] Zyto K, Ahrengart L, Sperber A, Törnkvist H (1997). Treatment of displaced proximal humeral fractures in elderly patients. J Bone Joint Surg Br.

[39] Kristiansen B, Kofoed H (1988). Transcutaneous reduction and external fixation of displaced fractures of the proximal humerus. A controlled clinical trial. J Bone Joint Surg Br.

[40] Kristiansen B, Kofoed H (1987). External fixation of displaced fractures of the proximal humerus. Technique and preliminary results. J Bone Joint Surg Br.

[41] Kristiansen B, Christensen SW (1986). Plate fixation of proximal humeral fractures. Acta Orthop Scand.

[42] Goldman RT, Koval KJ, Cuomo F, Gallagher MA, Zuckerman JD (1995). Functional outcome after humeral head replacement for acute three- and four-part proximal humeral fractures. J Shoulder Elbow Surg.

[43] Kumar GN, Sharma G, Sharma V, Jain V, Farooque K, Morey V (2014). Surgical treatment of proximal humerus fractures using PHILOS plate. Chin J Traumatol.

[44] Giannoudis PV, Xypnitos FN, Dimitriou R, Manidakis N, Hackney R (2012). Internal fixation of proximal humeral fractures using the Polarus intramedullary nail: our institutional experience and review of the literature. J Orthop Surg Res.

[45] Zhang L, Zheng J, Wang W (2011). The clinical benefit of medial support screws in locking plating of proximal humerus fractures: a prospective randomized study. Int Orthop.

[46] Lopiz Y, Garcia-Coiradas J, Garcia-Fernandez C, Marco F (2014). Proximal humerus nailing: a randomized clinical trial between curvilinear and straight nails. J Shoulder Elbow Surg.

[47] Sebastiá-Forcada E, Cebrián-Gómez R, Lizaur-Utrilla A, Gil-Guillén V (2014). Reverse shoulder arthroplasty versus hemiarthroplasty for acute proximal humeral fractures. A blinded, randomized, controlled, prospective study. J Shoulder Elbow Surg.

[48] Chalmers PN, Slikker W 3rd, Mall NA, Gupta AK, Rahman Z, Enriquez D, Nicholson GP (2014). Reverse total shoulder arthroplasty for acute proximal humeral fracture: comparison to open reduction-internal fixation and hemiarthroplasty. J Shoulder Elbow Surg.

[49] Garrigues GE, Johnston PS, Pepe MD, Tucker BS, Ramsey ML, Austin LS (2012). Hemiarthroplasty versus reverse total shoulder arthroplasty for acute proximal humerus fractures in elderly patients. Orthopedics.

[50] Olerud P, Ahrengart L, Ponzer S, Saving J, Tidermark J (2011). Internal fixation versus nonoperative treatment of displaced 3-part proximal humeral fractures in elderly patients: a randomized controlled trial. J Shoulder Elbow Surg.

[51] Seebach C, Nau C, Henrich D (2024). Cell-based therapy by autologous bone marrow-derived mononuclear cells for bone augmentation of plate-stabilized proximal humeral fractures: a multicentric, randomized, open phase IIa study. Stem Cells Transl Med.

[52] Fang C, Cai L, Chu G, Jarayabhand R, Kim JW, O'Neill G (2022). 3D printing in fracture treatment : Current practice and best practice consensus [Article in German]. Unfallchirurg.

[53] Combalia A, Sanchez-Vives MV, Donegan T (2024). Immersive virtual reality in orthopaedics—a narrative review. Int Orthop.

[54] Smith WR, Hudson PW, Ponce BA, Rajaram Manoharan SR (2018). Nanotechnology in orthopedics: a clinically oriented review. BMC Musculoskelet Disord.

[55] Hodgson S (2006). Proximal humerus fracture rehabilitation. Clin Orthop Relat Res.

[56] Howell J, Ayanian J (2016). Ernest Codman and the end result system: a pioneer of health outcomes revisited. J Health Serv Res Policy.

[57] Brinker MR, Hanus BD, Sen M, O'Connor DP (2013). The devastating effects of tibial nonunion on health-related quality of life. J Bone Joint Surg Am.

[58] Huskisson EC (1974). Measurement of pain. Lancet.

[59] Edelson G, Safuri H, Salami J, Vigder F, Militianu D (2008). Natural history of complex fractures of the proximal humerus using a three-dimensional classification system. J Shoulder Elbow Surg.

[60] Slobogean GP, Noonan VK, O'Brien PJ (2010). The reliability and validity of the Disabilities of Arm, Shoulder, and Hand, EuroQol-5D, Health Utilities Index, and Short Form-6D outcome instruments in patients with proximal humeral fractures. J Shoulder Elbow Surg.

[61] Tingart M, Bäthis H, Lefering R, Bouillon B, Tiling T (2001). Constant Score and Neer Score. A comparison of score results and subjective patient satisfaction [Article in German]. Unfallchirurg.

[62] Baker P, Nanda R, Goodchild L, Finn P, Rangan A (2008). A comparison of the Constant and Oxford shoulder scores in patients with conservatively treated proximal humeral fractures. J Shoulder Elbow Surg.

[63] Hudak PL, Amadio PC, Bombardier C (1996). Development of an upper extremity outcome measure: the DASH (disabilities of the arm, shoulder, and head). Am J Ind Med.

[64] Beaton DE, Wright JG, Katz JN (2005). Development of the QuickDASH: comparison of three item-reduction approaches. J Bone Joint Surg Am.

[65] Dawson J, Fitzpatrick R, Carr A (1996). Questionnaire on the perceptions of patients about shoulder surgery. J Bone Joint Surg Br.

[66] Richards RR, An KN, Bigliani LU (1994). A standardized method for the assessment of shoulder function. J Shoulder Elbow Surg.

[67] Lädermann A, Denard PJ, Collin P, Ibrahim M, Bothorel H, Chih-Hao Chiu J (2021). Single assessment numeric evaluation for instability as an alternative to the Rowe score. J Shoulder Elbow Surg.

[68] Baker HP, Gutbrod J, Strelzow JA, Maassen NH, Shi L (2022). Management of proximal humerus fractures in adults—a scoping review. J Clin Med.

[69] Calori GM, Colombo M, Bucci MS (2016). Complications in proximal humeral fractures. Injury.

[70] Kuo RY, Harrison C, Curran TA (2022). Artificial intelligence in fracture detection: a systematic review and meta-analysis. Radiology.

